# Blood pressure percentile charts to identify high or low blood pressure in children

**DOI:** 10.1186/s12887-016-0633-7

**Published:** 2016-07-19

**Authors:** Ashish Banker, Cynthia Bell, Monesha Gupta-Malhotra, Joshua Samuels

**Affiliations:** Division of Pediatric Cardiology, University of Texas McGovern Medical School at Houston / Children’s Memorial Hermann Hospital, TexasMedical Center, 6431 Fannin Street, MSB 3-121, Houston, 77030 TX USA; Divisions of Pediatric Nephrology & Hypertension, University of Texas McGovern Medical School at Houston / Children’s Memorial Hermann Hospital, Texas Medical Center, 6431 Fannin Street, MSB 3-121, Houston, 77030 TX USA

**Keywords:** Blood pressure, Chart, Graph, Stature, Hypertension, Hypotension, Pediatrics

## Abstract

**Background:**

The goal was to develop familiar blood pressure (BP) charts representing BP percentile curves similar to CDC growth charts to improve screening of both high and low BP in children.

**Methods:**

Since height accounts for substantially more BP variability than age and is a more direct measure of body size and maturation in children, height-specific BP percentile curves were drawn separately for males and females. We used the 2004 *Fourth Report* data source and equations to calculate the BP threshold value for each gender and 5 cm height group. By slightly underestimating a child’s BP percentile for high BP and slightly overestimating a child’s BP percentile for low BP, these charts guarantee 100 % sensitivity in detecting abnormal BP. Sensitivity and specificity of the chart cut-offs were confirmed in a sample of 1254 healthy children from a school-based blood pressure screening program.

**Results:**

The 1st, 5th, 25th, 50th, 75th, 90th, 95th, and 99th BP percentile curves are depicted in the chart for each corresponding gender and height from 85 to 190 cm, mimicking the ubiquitous CDC “growth charts”. Shaded areas of the chart differentiate abnormal BP status categories: hypotension, normal BP, prehypertension, Stage 1 hypertension, and Stage 2 hypertension. Sensitivity was confirmed to be 100 % with specificity above 94 %.

**Conclusions:**

These simplified BP charts improve upon currently available BP screening reference with the following features: (a) tracking BP longitudinally in an individual child, (b) full physiological range of BP percentiles represented in percentile curve format for rapid identification both high and low BP, (c) easy to use with absolute height alone avoiding the additional step of determining height percentile, (d) incorporation of adult threshold for pre-hypertension to assist in accurate transition from adolescence into adulthood, (e) high sensitivity and specificity to ensure all children at risk are identified with very few false positives.

## Background

Recognizing blood pressure (BP) abnormalities in children is more cumbersome than in adults and contributes to underdiagnoses in pediatrics [1–5]. While adult blood pressure thresholds are static, 140/90 mmHg for hypertension and 90/60 mmHg for hypotension, each child has his/her own BP thresholds based on gender, age, and height percentile [5]. While BP tracks with growth in children, similar to anthropometric measurements such as height and weight, it is not currently charted in the same way using growth charts [6]. Instead complex BP tables consisting of 476 threshold blood pressure values are used to lookup the BP percentile for a child [5]. Often these tables are used incorrectly or not at all [1].

The American Academy of Pediatrics has endorsed NHLBI guidelines that recommend all children over 3 years of age have BP measured at least annually for early recognition of BP abnormalities such as hypertension or syncope [7], although studies have shown this screening is rarely done in children. The implementation of a simple graphical representation of a child’s BP percentile similar to growth charts currently used for height, weight, BMI, and head circumference would improve and streamline pediatric primary care. Although several simplified BP charts have been reported in literature [8–15], the available US based charts are based on outdated reference values and use age as the primary body size reference. Height (stature), rather than age, is a better primary reference metric because it is a more precise measure of body size and maturation which are the primary determinants of the natural rise of BP throughout childhood [16–18]. Additionally, none of these published BP charts depict lower BP thresholds below the 50th percentile.

The purpose of this paper is to create a simple and practical BP screening tool to use alongside existing growth charts by the Center Of Disease Control (CDC) and World Health Organization (WHO) [6]. We create blood pressure charts based on BP thresholds derived from the *Fourth Report* which can be used to identify any BP percentile, including abnormally high or low pressures [5]. Because height accounts for substantially more BP variability than age in children [16–18], we will reference BP thresholds to absolute height in a gender-specific chart format.

## Methods

Although the study was approved by the local IRB (HSC-MS-12-0432), no subjects were required for the analysis so no informed consent was necessary. We used the 2004 *Fourth Report* as the source for all threshold values in our simplified charts. Current *Fourth Report* tables contain 476 blood pressure threshold values for each gender, age, and height percentile generated from regression model equations provided in the appendix of the *Fourth Report* [5]. To create our simplified blood pressure charts, we calculated the lowest blood pressure threshold value for each gender and 5-cm height group. This procedure was done for each high blood pressure percentile separately (75th, 90th, 95th, and 99th). Despite higher values defining the 90th percentile, the threshold value to define pre-hypertension was capped at 120/80 mmHg as suggested in the *Fourth Report*. For low blood pressure percentiles (25th, 10th, 5th, and 1st) the highest blood pressure threshold value for each gender and 5-cm height group was used. Median threshold value was used for the 50th blood pressure percentile. The range of age and height used in the equations were taken directly from the 2000 CDC stature-for-age tables for age in half-years and height percentiles 3rd, 5th, 10th, 25th, 50th, 75th, 90th, 95th, and 97th (www.cdc.gov/growthcharts). Only ages over 3 years were included as recommended by the *Fourth Report* and charts were created separately for boys and girls.

Our BP threshold values are direct summary measures of the thresholds calculated in the *Fourth Report* [5] such that minimum thresholds across ages are used to define hypertension and maximum thresholds across ages are used to define hypotension to make it a 100 % sensitive tool for screening. Stages of elevated blood pressure are shaded to delineate prehypertension (above 90th percentile or 120/80 mmHg and below 95th percentile), Stage 1 hypertension (above 95th percentile and below 99th percentile + 5 mmHg), and Stage 2 hypertension (above 99th percentile + 5 mmHg). Multiple pediatric critical care guidelines define hypotension to be less than the 5th percentile or less than 90/50 mmHg for children 10 years or older [19, 20]. Accordingly, the low blood pressure range (below 5th percentile and/or 90/50 mmHg) is shaded to identify hypotension.

We determined exact sensitivity and specificity estimates from a sample of 1254 healthy children whose blood pressure was measured as part of the University of Texas Houston Pediatric and Adolescent Hypertension Program (HPAHP) school-based blood pressure screenings. All children with blood pressure measured 4 times on 3 separate occasions with a Spacelabs 90217 oscillometric device at cross-sectional blood pressure screenings in 2011 and 2012 were included. We excluded children who reported use of hypertensive medication (*n* = 4), had missing BMI measurements (*n* = 6), or had missing age (*n* = 2). The 2nd, 3rd, and 4th average of blood pressures at the initial screen are used for classification by our simplified chart and compared to the gold standard *Fourth Report* thresholds: 95th percentile for hypertension and 90th percentile (or 120/80) for prehypertension. We did not estimate sensitivity or specificity estimates for hypotension since there is no gold standard for this diagnosis. Demographics were reported in mean (min- max), and count (%).

## Results

**Fig. 1:**
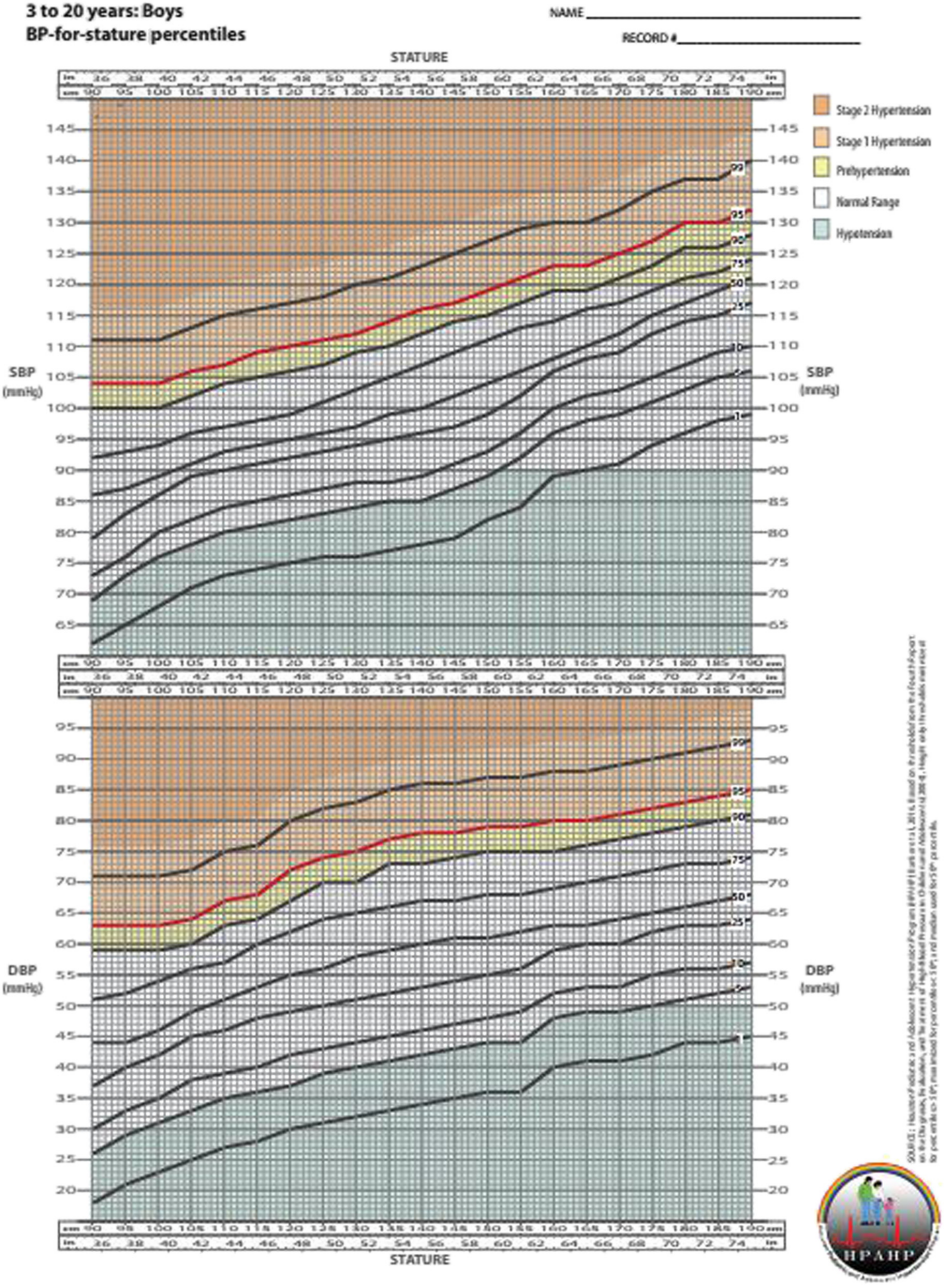
BP-for-stature percentiles, Boys 3 to 20 years (High definition BP Curve available at https://med.uth.edu/pediatrics/files/2013/07/BPChartBoyscolorwide.pdf)

**Fig. 2:**
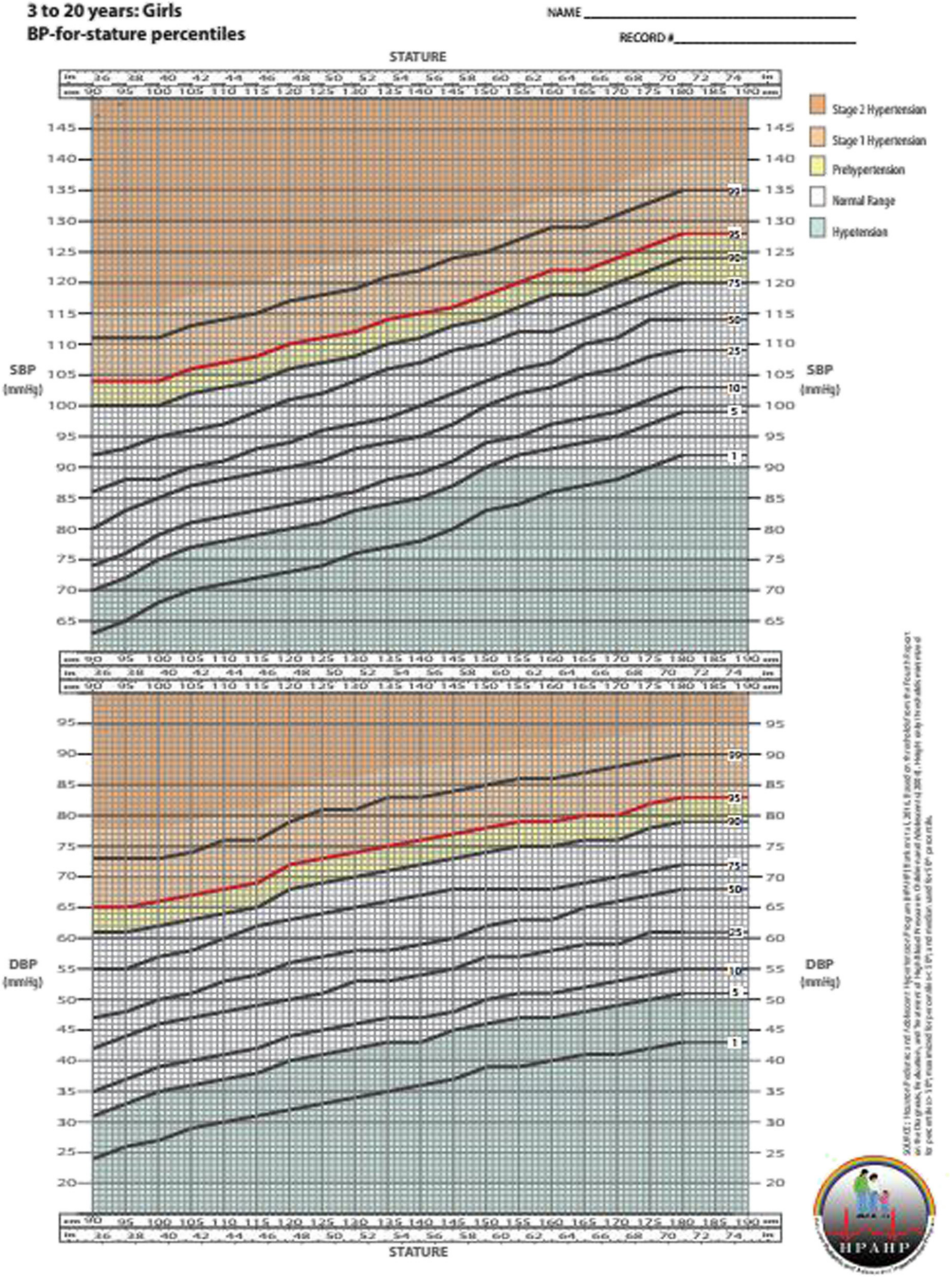
BP-for-stature percentiles, Girls 3 to 20 years (High definition Curve available at https://med.uth.edu/pediatrics/files/2013/07/BPChartGirlscolorwide.pdf)

Physiological BP charts with both systolic and diastolic percentiles are provided for boys (Fig. 1) and girls (Fig. 2). The BP charts are based on the height of the child scaled in metric units (cm) and English units (in) ranging from 85 to 190 cm. To assist the practitioner, color coded areas identifying abnormal high and low blood pressure are included in the BP charts.

We use these *Fourth Report* thresholds as the source for our simplified BP charts but display them in a 2-dimensional graphical format that are age-independent and require only gender and absolute height for reference. The calculation of height percentile is not required before using our BP charts as is with the *Fourth Report* tables.

We applied the simplified BP charts to a sample of 1254 healthy children with mean age 14.3 (10–19) years, BMI percentile 63.6 (0–100), height 158.0 (65.5–188.5) cm, 56.0 (21.5–143.2) kg, and 403 (32.1 %) females. The overall prevalence of hypertension was 103 (8.2 %) and prehypertension 143 (11.4 %) at the initial screening. The simplified BP charts identified 100 % of systolic and diastolic hypertension and prehypertension cases, as designed to maintain 100 % sensitivity (Tables 1 and 2). Systolic specificity was 94.7 % for hypertension and 95.4 % for prehypertension while diastolic specificity was higher at 99.3 % for hypertension and 98.3 % for prehypertension. Thus, false positive rates were controlled below 6 % for all high blood pressure categories.

**Table 1:** Classification of SBP in 1254 healthy children

		By Fourth Report table			
		Normal	Prehypertension	Stage 1 hypertension	Stage 2 hypertension
By simplified chart	Normal	967	0	0	0
	Prehypertension	45	81	0	0
	Stage 1 hypertension	2	59	73	0
	Stage 2 hypertension	0	0	15	12

**Table 2:** Classification of DBP in 1254 healthy children

		By Fourth Report table			
		Normal	Prehypertension	Stage 1 hypertension	Stage 2 hypertension
By simplified chart	Normal	1199	0	0	0
	Prehypertension	21	14	0	0
	Stage 1 hypertension	0	9	9	0
	Stage 2 hypertension	0	0	2	0

## Discussion

We present simplified two-dimensional BP charts for boys and girls sourced from current standard *Fourth Report* thresholds as a simple alternative to use in screening blood pressure in children above 3 years of age. According to the currently endorsed guidelines for children [5], six variables including systolic BP, diastolic BP, age, gender, height, and height percentile need to be determined in order to find upper BP thresholds from a detailed reference table [5] or lower BP thresholds from a different reference table or BP chart [19, 20]. Difficulties and inaccuracies using the current tables included in the *Fourth Report* have been documented in multiple studies. Bijlsma et al. recently showed that pediatric providers often fail to look up BP percentiles and thus frequently underestimate BP abnormalities [1]. Hansen et al. showed even when BP is measured, approximately 75 % of cases of hypertension and 90 % of cases of prehypertension in children and adolescents remain undiagnosed [2]. While efforts to create simpler and more practical tools to improve identification of hypertension in children have been suggested [21–24], none have become commonplace. Our charts alleviate the complexity of current BP classification by eliminating the need to determine height-percentile and by providing a quick visual representation of the child’s current BP percentile.

The decision to reference BP by gender and height alone instead of age was based on a careful historical analysis of the development of current *Fourth Report* guidelines. Originally, the 1987 Second Task Force Report [25] introduced only age- and gender- specific BP percentile curves as the first graphical representation of BP in children. Upon further investigation, height percentile was added to all subsequent updates because “body size is the most important determinant of BP in children and adolescents.” [5, 16] Additionally, height has been shown to be a better indicator of changes in BP than age and is independently related to BP in children [5, 17, 18]. Within any age, height can vary greatly resulting in a wide range of BP threshold values. Due to the methodological decision to use height percentile instead of absolute height in the *Fourth Report* polynomial regression equations, the difference in hypertension threshold between 5 and 95 % height percentiles is 9 mmHg in boys and 7 mmHg in girls for all ages [13]. Conversely, at any given height, age rarely varies by more than a few years, and hypertension threshold values are not as diffuse. For example, the shortest height for a 13 year old boy per the table is 56.5 in. and using this height value across different ages (9 through 13 years old) produces only a 2 mmHg difference in the 95th percentile systolic BP threshold.

Furthermore, BP-to-height ratio has been suggested as a practical and accurate metric in multiple studies across different populations and age groups as an alternative to using threshold tables [24, 26–29]. Although the BP-height ratio has potential, optimal-cut off points would need to be determined in children and validated in various populations before it is used for screening of elevated BP. The benefit of our BP charts is that the thresholds are based on the standard *Fourth Report* guidelines which have already been validated in many studies.

While the threshold values in the BP charts come directly from *Fourth Report* tables [5], the most conservative value for each height was used from a range of ages. Since, within a given height, the younger ages have a lower *Fourth Report* threshold, the largest deviations of 6 mmHg occur for children at the extremes of height-for-age (e.g., percentile <5th or >95th) [5]. This methodology of underestimating a child’s BP percentile for high BP and overestimating a child’s BP percentile for low BP guarantees 100 % sensitivity for detecting either low or high BP. Although this methodology results in slight variation from the *Fourth Report* thresholds [5], our BP charts are created for use as a screening tool rather than diagnosis. As a screening tool, sensitivity should be favored over specificity in order to avoid under diagnosis. Nevertheless, these simplified BP charts maintain a low false positive rate less than 6 % as seen with specificities above 94 %. When a child’s BP falls outside this normal range, repeat measurements should be made according to the *Fourth Report* guidelines [5] and a specialist consulted for hypertension or hypotension.

Currently no BP charts exist for use in children that are based on current *Fourth Report* thresholds and none include both upper and lower BP limits. Currently in pediatrics, definitive values for the diagnosis of systolic or diastolic hypotension are lacking. Most of the current guidelines used by the Pediatric Advanced Life Support (PALS) course [30], Brain Trauma Foundation (BTF) [31], and International Pediatric Sepsis Consensus Conference [32] define hypotension in children as systolic BP below 5th percentile for age reportedly derived from the *Fourth Report* data [19]. The inclusion of all percentiles in one chart will ease practitioners by facilitating referencing only this one image to quickly screen for both hypertensive and hypotensive BP abnormalities. While electronic medical record (EMR) systems, smartphones, and online calculators have eased the assessment of BP based on the Fourth Report tables [5], these technological tools require immediate point of care availability in clinical settings and are frequently underutilized. None allows for longitudinal tracking of BP percentile. To accommodate all situations, our simplified BP charts can be printed on paper (in color or grayscale) for use in low-resource clinical settings or incorporated into electronic formats for use in EMR systems.

## Conclusions

In summary, we provide simple BP charts for children representing percentile curves similar to CDC growth charts for rapid detection of both high and low BP. These BP charts parallel standard growth charts and improve upon current BP threshold resources with the following features: (a) tracking BP at various ages in the same child, (b) full physiological range of BP percentiles represented in curve format for rapid identification both high and low BP, (c) easy to use with absolute height alone avoiding the additional step of determining height percentile, (d) incorporation of adult threshold for pre-hypertension to assist in accurate transition from adolescence into adulthood, and (e) high sensitivity and specificity to ensure all children at risk are identified with very few false positives.

## Abbreviations

BP, blood pressure; HTN, hypertension
